# High Glucose and Carbonyl Stress Impair HIF-1-Regulated Responses and the Control of Mycobacterium tuberculosis in Macrophages

**DOI:** 10.1128/mbio.01086-22

**Published:** 2022-09-19

**Authors:** Graciela Terán, Hanxiong Li, Sergiu-Bogdan Catrina, Ruining Liu, Susanna Brighenti, Xiaowei Zheng, Jakob Grünler, Susanne Nylén, Berit Carow, Martin E. Rottenberg

**Affiliations:** a Department of Microbiology, Tumor and Cell Biology (MTC), Karolinska Institute t, Stockholm Sweden; b Department of Molecular Medicine and Surgery, Karolinska Institute t, Rolf Luft Research Center for Diabetes and Endocrinology, Karolinska University Hospital, Stockholm, Sweden; c Center for Diabetes, Academic Specialist Center, Stockholm, Sweden; d Center for Infectious Medicine (CIM), Department of Medicine, Karolinska Institute t, Stockholm, Sweden; Harvard School of Public Health

**Keywords:** diabetes, HIF-1, macrophage, *Mycobacterium tuberculosis*

## Abstract

Diabetes mellitus (DM) increases the risk of developing tuberculosis (TB), but the mechanisms behind diabetes-TB comorbidity are still undefined. Here, we studied the role of hypoxia-inducible factor-1 (HIF-1), a main regulator of metabolic and inflammatory responses, in the outcome of Mycobacterium tuberculosis infection of bone marrow-derived macrophages (BMM). We observed that M. tuberculosis infection of BMM increased the expression of HIF-1α and HIF-1-regulated genes. Treatment with the hypoxia mimetic deferoxamine (DFO) further increased levels of HIF-1-regulated immune and metabolic molecules and diminished the intracellular bacterial load in BMM and in the lungs of infected mice. The expression of HIF-1-regulated immunometabolic genes was reduced, and the intracellular M. tuberculosis levels were increased in BMM incubated with high-glucose levels or with methylglyoxal (MGO), a reactive carbonyl compound elevated in DM. In line with the *in vitro* findings, high M. tuberculosis levels and low HIF-1-regulated transcript levels were found in the lungs from hyperglycemic *Lepr^db/db^* compared with wild-type mice. The increased intracellular M. tuberculosis growth and the reduced expression of HIF-1-regulated metabolic and inflammatory genes in BMM incubated with MGO or high glucose were reverted by additional treatment with DFO. *Hif1a*-deficient BMM showed ablated responses of immunometabolic transcripts after mycobacterial infection at normal or high-glucose levels. We propose that HIF-1 may be targeted for the control of M. tuberculosis during DM.

## INTRODUCTION

A quarter of the global population has been infected with Mycobacterium tuberculosis. However, only a fraction of infected individuals develops tuberculosis (TB). Still, in 2020, 10 million people developed the disease and 1.5 million died from TB (1). Why some individuals develop TB is not completely understood. However, human beings with a compromised immune system, such as people living with HIV, have a higher risk of developing TB.

Infection occurs when inhaled M. tuberculosis reaches the lung alveoli and is phagocytized by resident alveolar macrophages (2). Infected macrophages or those activated by bacterial molecules will recruit mononuclear phagocytes. A mantle of lymphocytes will then surround these phagocytes, forming a granuloma, in which bacterial growth is either restricted or disseminated to other organs and can be transmitted to uninfected individuals.

Type 2 diabetes (DM) is a metabolic disease characterized by high blood-glucose levels secondary to an inappropriate insulin secretion for peripheral insulin sensitivity. Inflammation secondary to infection or obesity worsens metabolic control secondary to an increase in insulin resistance. Different cell types cannot decline their glucose uptake when exposed to hyperglycemia leading to high intracellular glucose levels (3), resulting in increased synthesis of reactive carbonyl compounds, such as methylglyoxal (MGO), a by-product of glycolysis. MGO mediates rapid nonenzymatic glycation of proteins, lipids, and DNA to promote the formation of advanced glycation end products (AGEs), which eventually renders irreversible damage to these macromolecules, including their integrity of structure and function (4). MGO and MGO-derived AGE thus impact tissue and organ functions and are important factors in vascular complication development in DM (5).

Epidemiologic studies have revealed a 3-fold higher risk of active TB among patients with DM than among individuals without DM and a strong association between TB and DM regardless of TB endemicity (6–8). About 15% of TB cases globally are linked to DM (9). While people with DM are at higher risk of progressing from latent to active TB, TB might also contribute to the worsening of metabolic control in subjects with DM (10). An exacerbated immunopathology is a frequent observation in TB that is complicated by DM (11). However, the underlying mechanisms behind the DM-TB association are largely unknown.

The adaptive response of mammalian cells to the stress of oxygen depletion is coordinated by the action of hypoxia-inducible transcription factors (HIFs). The alpha subunits of these transcription factors serve as the central sensor of oxygen tension in cells. HIFs function is regulated at least in part at the protein level via the degradation of the HIF-α subunits under normoxic conditions. The hydroxylation of proline residues of HIF-α is executed by prolyl-hydroxylases (PHDs) that use Fe^2+^, oxygen, and oxoglutarate as cofactors (12–14). The hydroxylated form of HIF-α binds to the von Hippel-Lindau (VHL) tumor suppressor protein that is part of an E3 ubiquitin ligase complex that targets HIF-α for proteasomal degradation (12, 15). Under hypoxia HIF-α, prolyl hydroxylation is inhibited, and HIF accumulates in the nucleus and transactivates HIF-responsive genes (16).

A number of isoforms of the α-subunit have been identified, but the main important functionality is HIF-1α and HIF-2α with both distinct and overlapping biological roles. HIF-1 has been shown to induce apoptotic pathways and drive the expression of genes that are involved in the glycolytic pathway, whereas HIF-2 preferentially promotes growth and angiogenesis (17, 18). HIFs are highly relevant to the proper function of different immune cell populations, including macrophages (19). Macrophages activated by microbial infection or by several innate receptor agonists switch their metabolism from oxidative phosphorylation to glycolysis, a response that is similar to the response to hypoxia. HIF-1 contributes to the expression of genes associated with macrophage activation (19, 20). Some of these HIF-1-regulated immune molecules, such as inducible nitric oxide synthase (iNOS) or interleukin-1β (IL-1β), have central roles in the intracellular control of M. tuberculosis (21, 22). HIF-1 has been shown to accumulate in the human hypoxic TB granuloma (23), and TB lesions in experimental animals show an increase in glycolytic pathways with a reduction of the tricarboxylic acid cycle and oxidative phosphorylation (24). Mice deficient in HIF-1 in myeloid cells showed higher susceptibility to infection with M. tuberculosis (25).

In the lung of infected individuals, M. tuberculosis-specific T cells will recognize infected macrophages and secrete interferon-γ (IFN-γ), required for macrophage activation, further inducing iNOS expression and containment of infection (26–28). Moreover, HIF-1 was shown to regulate IFN-γ responses of M. tuberculosis-infected macrophages; however, *hif1a*-deficient macrophages permitted the growth of M. tuberculosis even when activated with IFN-γ (25, 29).

Hyperglycemia has been shown to repress HIF-1 function (30), and a defective reaction of tissues to hypoxia due to HIF-1 inhibition has been suggested to be a pathogenetic mechanism in DM (31, 32).

Here, we hypothesize that an impaired HIF-1 function during DM would aggravate M. tuberculosis infection in mice by hampering protective immune responses. We showed that bone marrow-derived macrophages (BMM) express high levels of HIF-1-regulated genes after infection with M. tuberculosis or M. bovis BCG. Treatment with the hypoxia mimic deferoxamine (DFO) increased immunometabolic responses in infected BMMs and in the lungs of M. tuberculosis-infected mice. DFO treatment reduced the M. tuberculosis titers in BMM treated or not with IFN-γ and decreased the bacterial load in the lungs of infected mice. The incubation with either MGO or high-glucose concentrations hampered the expression of HIF-1-regulated genes and impaired bacterial control in M. tuberculosis-infected BMM. Treatment with DFO restored HIF-1-regulated responses to infection and improved M. tuberculosis control in MGO and high-glucose-treated cells.

## RESULTS

### HIF-1-dependent responses increase in mycobacteria-infected BMM and in lungs of M. tuberculosis-infected mice.

**FIG 1:**
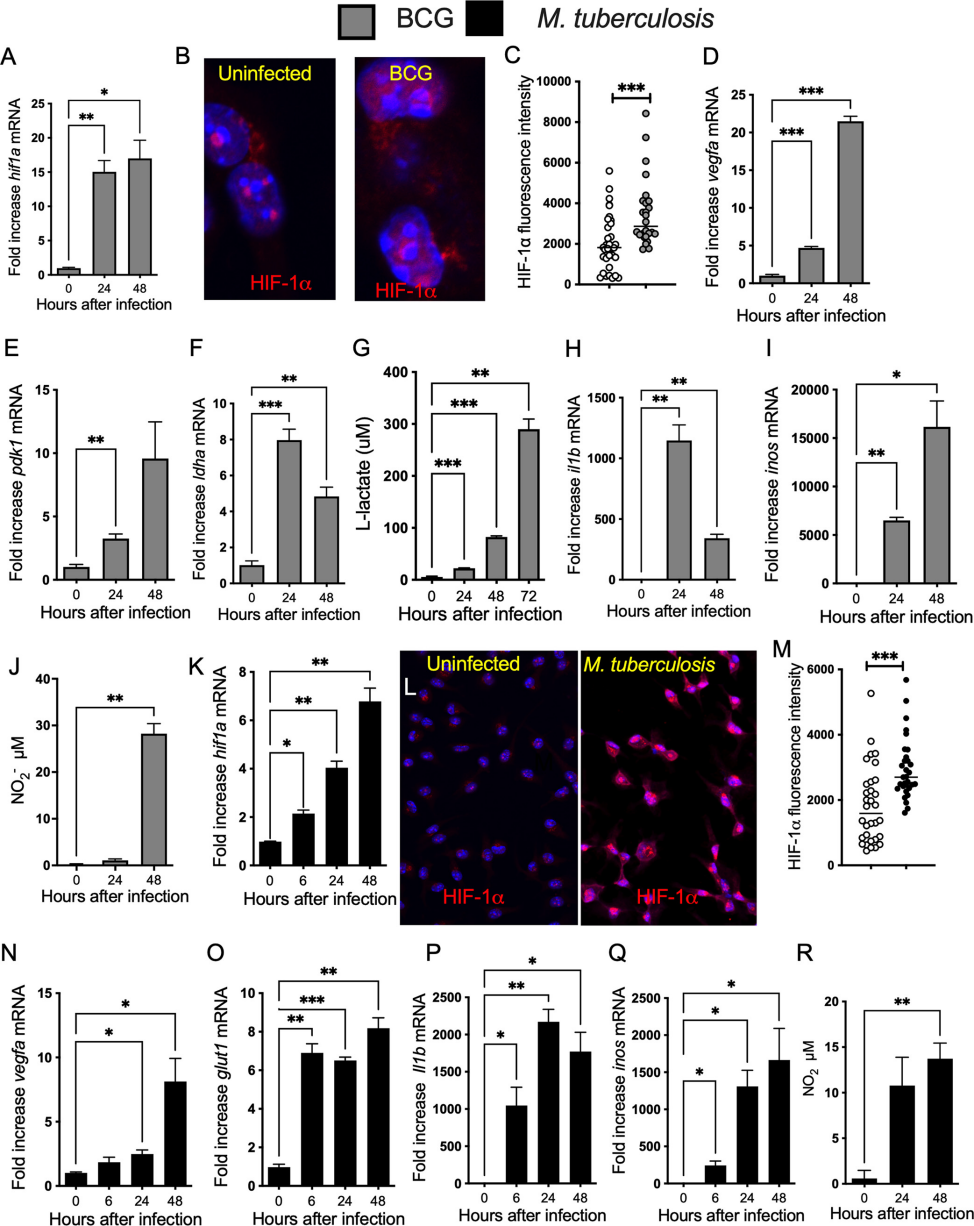
HIF-1-regulated responses increase in mycobacteria-infected BMM. (A, D to F, H, I, K, and N to Q) Total RNA was extracted from triplicate independent cultures of BMM before and at different time points after infection with BCG (A, D to F, H, and I) or M. tuberculosis (K and N to Q) at an MOI of 5:1. The relative concentration of *hif1a* (A and K), *vegfa* (D and N), *pdk1* (E), *ldha* (F), *glut1* (O), *il1b* (H and P), and *inos* (I and Q) transcripts in relation to *hprt* mRNA levels in the same sample was determined by real-time PCR. Differences with noninfected controls are significant at *, *P* ≤ 0.05; **, *P* ≤ 0.01; and ***, *P* ≤ 0.001, Student's *t* test. (B, C, L, and M) Micrographs showing labeling for HIF-1α in BMM 24 h after either BCG (B) or M. tuberculosis (I) infection and in uninfected controls (DAPI was used for nuclear staining). The HIF-1α label intensity in BMM 24 h after infection with BCG (C) or M. tuberculosis (M) infected BMM was quantified using the Cell Profiler software. The quantification was performed in 3 independent samples (4 field of view per sample). The individual and the mean HIF-1α levels per cell in one field of view are shown. Differences are significant at ***, *P* ≤ 0.001, unpaired Student's *t* test. (G) The lactate concentration accumulated in the supernatants from BMM infected with M. bovis BCG (MOI 1:1) was measured by lactate dehydrogenase assay. The data represent the results of triplicate independent cultures ± SEM. A representative of 2 experiments is shown. Differences with noninfected controls are significant at **, *P* ≤ 0.01 and ***, *P* ≤ 0.001, unpaired Student's *t* test. (I and R) The concentration of nitrite in the supernatants of either M. tuberculosis (I) or BCG-infected (R) BMM was measured at different times after infection. The mean levels of NO_2_^−^ ± SEM in independent triplicates were measured by Griess assay.

We first analyzed the expression of HIF-dependent responses during infection of BMM with mycobacteria. The expression of *hif1a* mRNA and HIF-1α protein was enhanced in the attenuated M. bovis BCG-infected BMM (Fig. 1A to C and Fig. S1A in the supplemental material). The expression levels of HIF-1-regulated *vegfa* and the glycolytic transcripts *pdk1* and *ldha* mRNA were also increased in M. tuberculosis-infected BMM (Fig. 1D to F). In line with this, lactate, the final product of glycolysis was increased in BCG-infected BMM (Fig. 1G). HIF-1 has been shown to induce *il1b* and *inos* expression and increase nitric oxide (NO) production (20, 33), and *il1b* and *inos* mRNA and the nitrite concentration were increased in BMM at different times after infection with BCG (Fig. 1H to J). The infection of BMM with virulent M. tuberculosis also increased the levels of HIF-1α mRNA and protein expression (Fig. 1K to M). HIF-1 regulated glycolytic transcripts (Fig. 1N and O) and *il1b* and *inos* transcripts (Fig. 1P and Q), and the levels of nitrite (Fig. 1R) were all increased in M. tuberculosis-infected BMM.

10.1128/mbio.01086-22.1 FIG S1 HIF-1-regulated responses increase in lungs from M. tuberculosis-infected mice. (A) Micrographs showing labeling for HIF-1α in BMM 24 h after either BCG and in uninfected controls (40× lens objective). (B to D) Groups of C57BL/6 mice were infected with 200 M. tuberculosis via aerosol route. The total RNA was extracted from lungs at the indicated days after infection (*n* = 4 per time point). The levels of *hif1a* (B)*, glut1* (C), and *vegfa* (D) mRNA were determined by real-time PCR as indicated above. Differences with the untreated group are significant at *, *P* ≤ 0.05 and **, *P* ≤ 0.01, one-way ANOVA with Welch’s correction. Download FIG S1, EPS file, 2.1 MB. Copyright © 2022 Terán et al. 2022 Terán et al. https://creativecommons.org/licenses/by/4.0/ This content is distributed under the terms of the Creative Commons Attribution 4.0 International license.

The expression of HIF-1-regulated metabolic genes was then evaluated in the lungs of C57BL/6 mice infected with M. tuberculosis via the aerosol route. We found that levels of *hif1a*, *glut1*, and *vegfa* mRNA were all elevated after infection with M. tuberculosis (Fig. S1B and D).

### DFO enhances HIF-1-regulated responses and M. tuberculosis control in BMM.

**FIG 2:**
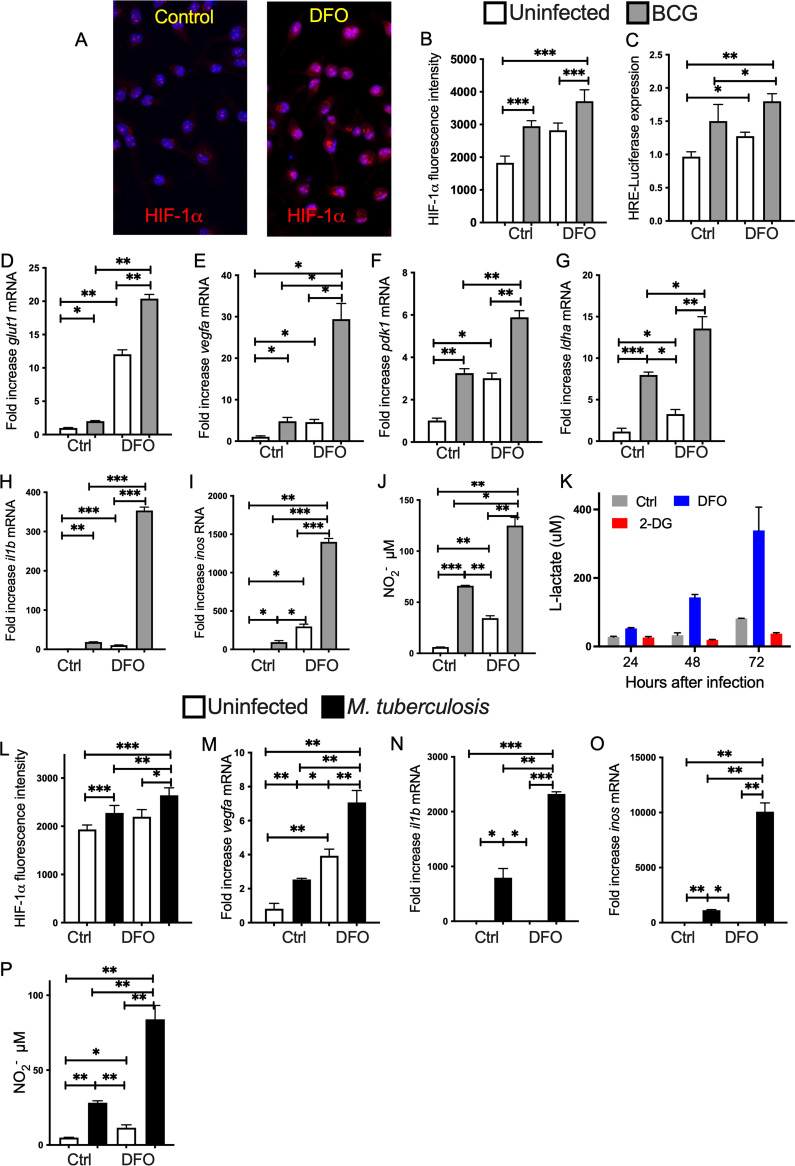
DFO stimulates HIF-1-mediated immune and metabolic responses in BCG and M. tuberculosis-infected BMM. (A and B) BMM were infected with BCG and treated with 100 μM DFO starting 4 h after infection or left untreated. (A) The micrograph of HIF-1α immunolabeling 24 h after DFO treatment in noninfected cultures. The fluorescence intensity of HIF-1α was determined in triplicate independent cultures (4 determinations per slide). (B) The mean HIF-1α intensity per cell ± SEM in one representative sample is shown. Differences are significant at *, *P* ≤ 0.05; **, *P* ≤ 0.01; and ***, *P* ≤ 0.001, one-way ANOVA with Welch’s correction. (C) The relative HRE activity in RAW macrophages treated or not with DFO was evaluated 24 h after BCG infection. The mean relative luciferase levels ± SEM are in triplicated cultures are depicted. One of two experiments is depicted. Differences are significant at *, *P* ≤ 0.05; **; *P* ≤ 0.01; and ***, *P* ≤ 0.001 001, one-way ANOVA with Welch’s correction. (D to I) Total RNA was extracted 24 h after treatment and the levels of *glut1* (D), *vegfa* (E), *pdk1* (F), *ldha* (G), *il1b* (H), and *inos* (I) mRNA were measured by real-time PCR. The mean fold increase of mRNA concentration in triplicate cultures ± SEM compared to an uninfected and untreated control ± SEM is depicted. One out of at least 3 experiments is depicted. Differences are significant at **, *P* ≤ 0.01 and ***, *P* ≤ 0.001, one-way ANOVA test with Welch’s correction. (J) The concentration of nitrite in the supernatants of BCG-infected and or DFO-treated BMM was measured 48 h after culture. The mean levels of NO_2_^−^ ± SEM in independent triplicates was measured by Griess assay. Differences are significant at **, *P* ≤ 0.01 and ***, *P* ≤ 0.001, one-way ANOVA test with Welch’s correction. (K) The lactate concentration was measured in the supernatants of BMM cultures after treatment with either 100 μM DFO or 300 μM 2-DG at different times after BCG infection. (L) HIF-1α was labeled in BMM infected with M. tuberculosis and treated with 100 μM DFO starting 4 h after infection or left untreated. The mean HIF-1α intensity per cell ± SEM in one representative of 3 independent samples is shown. Differences are significant at *, *P* ≤ 0.05; **, *P* ≤ 0.01; and ***, *P* ≤ 0.001 one-way Welch’s test ANOVA. (M to O) The fold increase of *vegfa* (M), *il1b* (N), and *inos* (O) transcripts in BMM cultures 24 h after M. tuberculosis infection treated or not with DFO as indicated above (*n* = 3 per group) ± SEM are depicted. Differences are significant at *, *P* ≤ 0.05; **, *P* ≤ 0.01; and ***, *P* ≤ 0.001, one-way Welch’s ANOVA test. (P) The concentration of nitrite in the supernatants of M. tuberculosis-infected and or DFO-treated BMM was measured 72 h after culture. Differences are significant at *, *P* ≤ 0.05 and **, *P* ≤ 0.01 one-way Welch’s ANOVA test.

DFO is an iron chelator that stabilizes HIFs by inhibiting PHDs (34). Treatment of BMM with 100 μM DFO for 24 h increased the expression of HIF-1α (Fig. 2A and B). Using a HIF-1 reporter assay, we observed that DFO increased HIF-1 activity in macrophages before or after infection with BCG (Fig. 2C). BCG-infected BMM were treated with 100 μM DFO 4 h after infection. The concentration of *glut1*, *vegf*, *pdk1*, and *ldha* mRNAs in uninfected BMM was increased after DFO treatment (Fig. 2D to G) and DFO further increased levels of these transcripts in BCG-infected BMM (Fig. 2D to G). *il1b* and *inos* mRNAs (Fig. 2H and I) and nitrite levels in the culture supernatants (Fig. 2J) were also increased after DFO treatment of infected BMM. DFO stimulation of uninfected cells resulted in low *il1b*, *inos* mRNA, and nitrite in supernatants compared to that of mycobacteria-infected BMM (Fig. 2H to J). The levels of lactate were also elevated in supernatants of mycobacteria-infected BMM treated with DFO, whereas coincubation with 2-DG, an inhibitor of glycolysis, reduced the lactate levels in culture supernatants (Fig. 2K). DFO treatment of M. tuberculosis-infected BMM also increased the levels of HIF-1α (Fig. 2L) and metabolic and inflammatory transcripts (Fig. 2M to O), as well as nitrite levels in the culture supernatants (Fig. 2P).

We next studied, whether DFO also increased the levels of HIF-1-regulated transcripts *in vivo*. For this purpose, mice were administered intraperitoneally (i.p.) with 400 mg/kg DFO every other day and sacrificed the day after the last dose. The levels of *glut1* and *vegfa* mRNA in lungs, spleens, and livers were elevated in DFO-treated mice (Fig. S2A, B, D, E, and H). In addition, the levels of *inos* mRNA in livers, but not lungs and spleens, of DFO-treated mice were found to be increased (Fig. S2C, F, and I).

10.1128/mbio.01086-22.2 FIG S2 Administration of mice DFO increases the titers of HIF1-α-regulated transcripts. (A to I) C57BL/6 mice were inoculated i.p. with 400 mg/kg DFO, received 3 doses every other day, and sacrificed a day after the last dose. The mean relative levels of *vegfa* (A, D, and G), *glut1* (B, E, and H), and *inos* (C, F, and I) transcripts in lungs (A to C), spleens (D to F), and livers (G to I) ± SEM (*n* = 5 per group) from treated or control mice were measured by real-time PCR. Differences with the untreated group are significant at *, *P* ≤ 0.05 and **, *P* ≤ 0.01, unpaired Student’s *t* test. Download FIG S2, EPS file, 0.4 MB. Copyright © 2022 Terán et al. 2022 Terán et al. https://creativecommons.org/licenses/by/4.0/ This content is distributed under the terms of the Creative Commons Attribution 4.0 International license.

**FIG 3:**
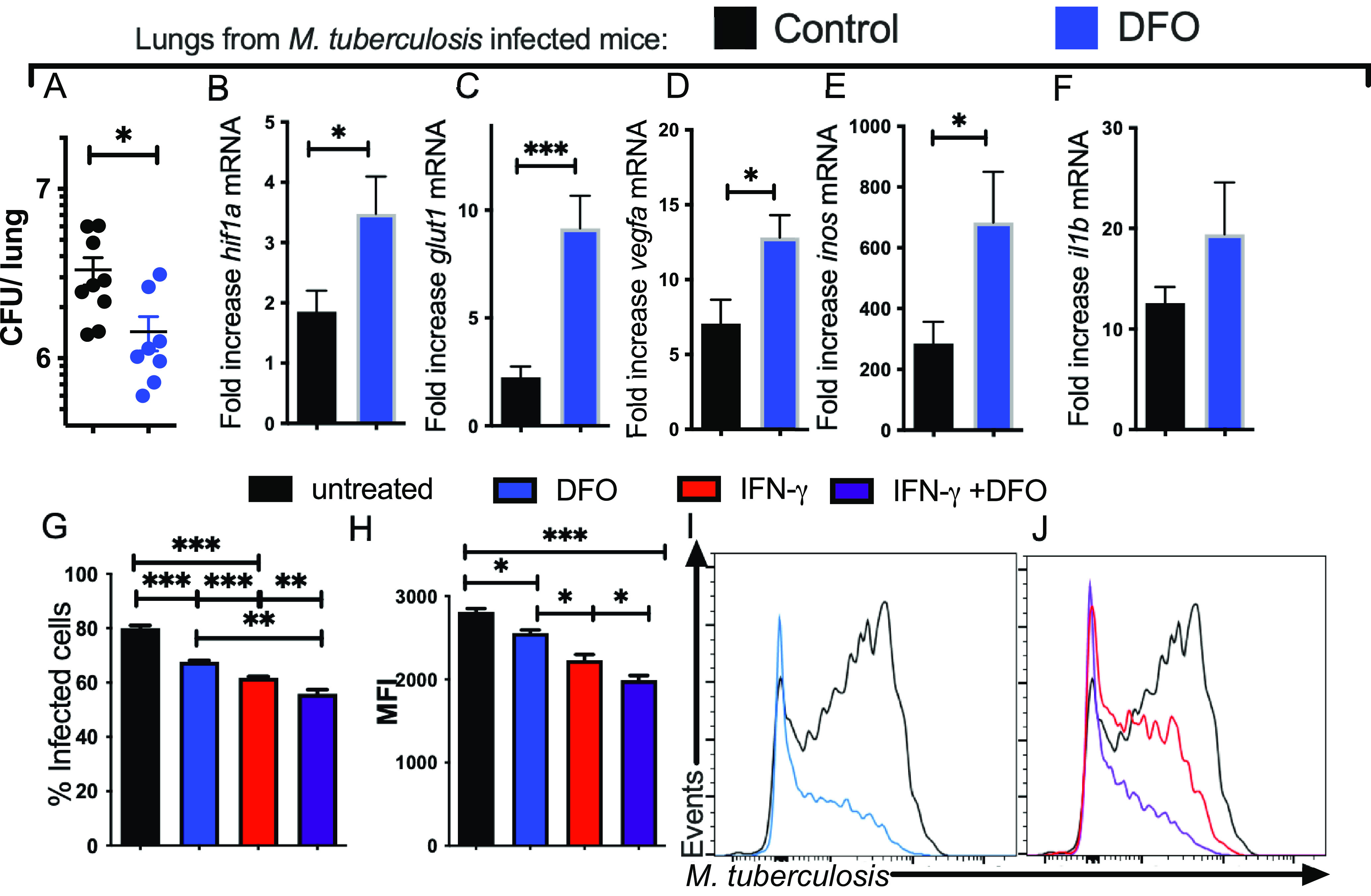
DFO treatment of BMM improves the intracellular growth control of M. Tuberculosis. (A) C57BL/6 mice were treated with 400 mg/kg DFO i.p. every other day after aerosol infection with M. tuberculosis for 12 weeks. The group median and the individual CFU per lung of DFO-treated and control mice (*n* = 9 per group) are depicted (median CFU/lung: control 2.80·10^6^; DFO treated: 7·10^6^). Differences in CFU between DFO-treated and untreated groups are significant (*, *P* < 0.05, Mann-Whitney U test). (B to F) The mean relative levels ± SEM of *hif1a* (B), *glut1* (C), *vegfa* (D), *inos* (E), and *il1b* (F) transcripts in lungs of DFO-treated or control mice (*n* = 5 per group) were measured by real-time PCR. The results were normalized with the level of a transcript in the lung of an untreated and uninfected control animal. Differences with the untreated group are significant at *, *P* ≤ 0.05 and ***, *P* ≤ 0.001, Student's *t* test with Welch’s correction. (G to J) A group of BMM was treated with IFN-γ starting 48 h before M. tuberculosis*-*gfp infection (MOI 1:1). Groups of IFN-γ-activated and control BMM were incubated with DFO 4 h after infection or left untreated. The mean percentage of infected BMM (G) and the mean M. tuberculosis-gfp MFI gated on infected cells (H) determined 5 days after infection and representative histograms are shown (I and J). Differences are significant at *, *P* ≤ 0.05; **, *P* ≤ 0.01; and ***, *P* ≤ 0.001, one-way ANOVA test with Welch’s correction.

Then, we investigated whether DFO administration every other day for 3 months altered the outcome of M. tuberculosis-aerosol infection in mice. We found that DFO-treated mice showed reduced levels of M. tuberculosis in lungs (Fig. 3A). In line with these results, levels of *hif-1a*, *vegfa*, *glut1*, and *inos* mRNA were elevated in the lungs of M. tuberculosis-infected, DFO-treated mice (Fig. 3B to E) compared to DFO-untreated, -infected controls. Levels of *il1b* showed a trend toward increased levels without reaching statistical significance (Fig. 3F).

The effect of DFO in the control of intracellular M. tuberculosis growth was then tested. The intracellular levels of green fluorescent protein (gfp)-expressing M. tuberculosis and the frequency of infected cells increased with the multiplicity of infection (MOI) used and with the time after infection, as also observed when evaluating M. tuberculosis infection on adherent BMM (Fig. S3A to F). Preincubation of cells with IFN-γ did not alter the bacterial uptake when measured 4 h after infection (Fig. S3G) but reduced the levels of intracellular M. tuberculosis and the percentage of infected BMM when measured 5 days after infection (Fig. S3H). The incubation of BMM with DFO reduced the intracellular load and percentage of infected cells 5 days after infection compared to controls (Fig. 3G to I). Moreover, the culture of BMM with both DFO and IFN-γ further reduced the intracellular M. tuberculosis load compared to BMM treated with either IFN-γ or DFO alone (Fig. 3G to I). DFO did not hamper the growth of M. tuberculosis in axenic cultures at the concentrations used in the BMM cultures (Fig. S3I).

10.1128/mbio.01086-22.3 FIG S3 Intracellular growth of M. tuberculosis in bone marrow-derived macrophages. (A) Representative histograms of M. tuberculosis-gfp infected BMM at different MOI measured 4 or 120 h after infection with different MOI are shown. (B and C) The mean percentage of infected BMM ± SEM (B) and the mean M. tuberculosis-gfp MFI ± SEM (C) at different times after infection with different MOIs are depicted. (D to F) Representative micrographs of adherent BMM using different MOIs and determined 4 and 72 h after M. tuberculosis-gfp infection. Red, falloidin; blue, DAPI; and green, M. tuberculosis-gfp. (E and F) The mean % of infected BMM (E) and the mean fluorescent intensity of M. tuberculosis-gfp infected BMM (F) were determined on slides. (G and H) Representative histograms of BMM stimulated or not with IFN-γ 48 h before M. tuberculosis-gfp infection MOI1:1 and MOI 3:1; the M. tuberculosis*-*gfp levels are shown at 4 h (MOI 1:1) (G) and 120 h (H) after infection. (I) The growth kinetics of M. tuberculosis in axenic cultures containing different DFO concentrations. Download FIG S3, TIF file, 0.7 MB. Copyright © 2022 Terán et al. 2022 Terán et al. https://creativecommons.org/licenses/by/4.0/ This content is distributed under the terms of the Creative Commons Attribution 4.0 International license.

### MGO hampers HIF-1 responses and the growth control M. tuberculosis in BMM.

**FIG 4:**
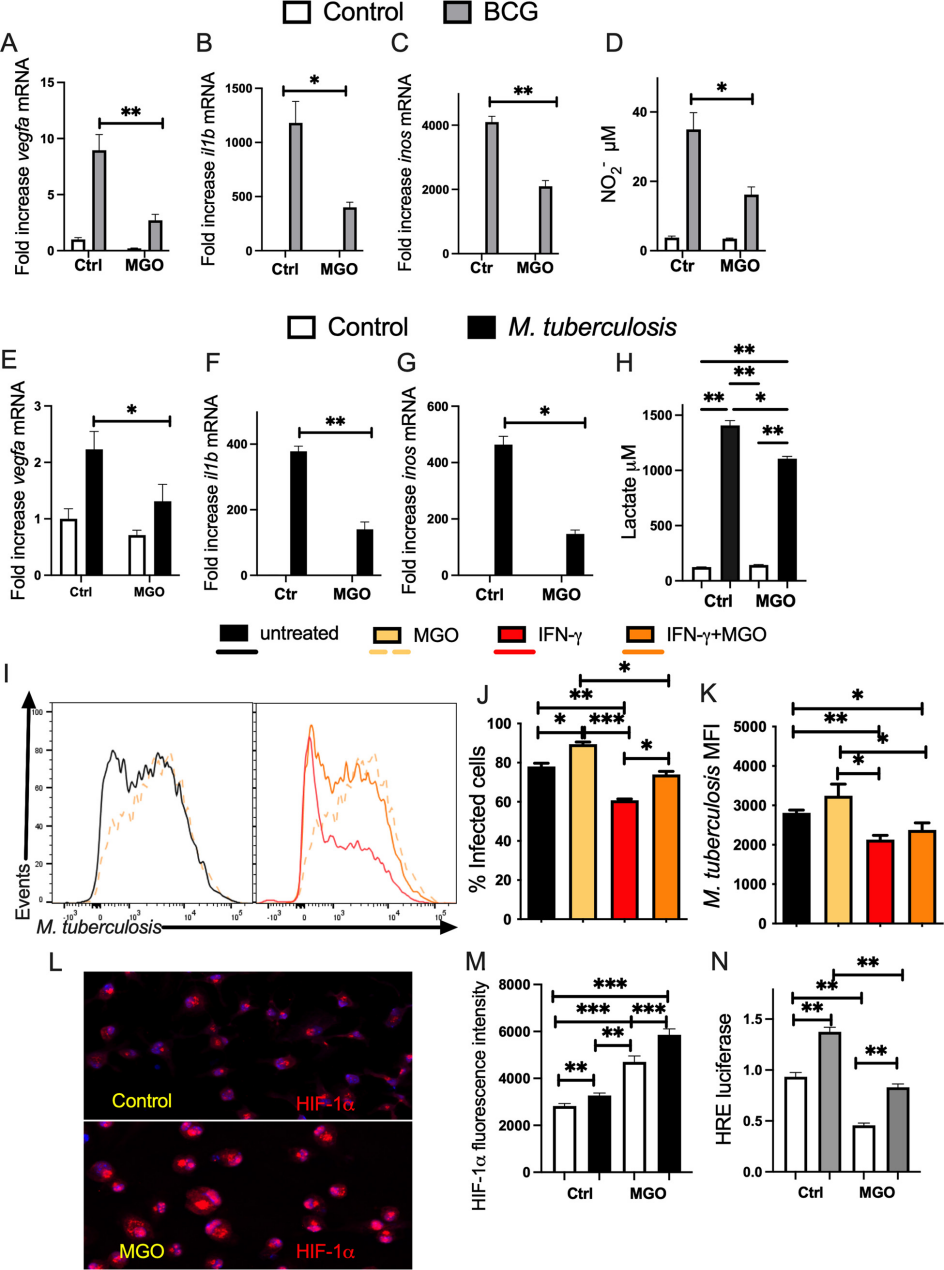
Treatment with methylglyoxal (MGO) hampers control of M. tuberculosis infection and HIF-1-regulated responses of BMM A-C.BMM were incubated with 200 μM MGO and infected with BCG 4 h after. (A to C) The mean fold increase of *vegfa* (A), *il1b* (B), and *inos* (C) mRNA was measured in triplicate BMM cultures ± SEM as measured by RT-PCR. Differences are significant at *, *P* ≤ 0.05 and **, *P* ≤ 0.01, one-way ANOVA with Welch’s adjustment. (D) The concentration of nitrite in the supernatants of MGO-treated and/or BCG-infected BMM was measured 72 h after culture. (E to G) BMM incubated with 125 μM MGO for 4 h and infected with M. tuberculosis thereafter. The mean fold increase of *vegfa* (E), *il1b* (F), and *inos* (G) mRNA was measured in triplicate BMM cultures ± SEM was measured by RT-PCR. Differences between infected groups are significant at *, *P* ≤ 0.05 and **, *P* ≤ 0.01, one-way ANOVA with Welch’s adjustment. (H) The lactate concentration in the supernatants from BMM treated with MGO 4 h before infection with M. tuberculosis was measured by lactate dehydrogenase assay. The mean lactate concentration ± SEM in BMM supernatants 72 h after infection is shown. Differences are significant at *, *P* ≤ 0.05 and **, *P* ≤ 0.01, one-way ANOVA with Welch’s adjustment. (I) A group of BMM was treated with IFN-γ starting 48 h before M. tuberculosis*-*gfp infection. IFN-γ-treated and control BMM were treated with 400 μM MGO 4 h before infection with M. tuberculosis*-*gfp (MOI 1:1). (I to K) Representative histograms (I), the mean percentage of infected BMM (J), and the MFI of intracellular M. tuberculosis (K) quantified 5 days after infection are shown. Differences are significant at *, *P* ≤ 0.05; **, *P* ≤ 0.01; and ***, *P* ≤ 0.001, one-way ANOVA with Welch’s correction. (L and M) Representative micrographs (L) of uninfected MGO-treated and control BMM and the mean ± SEM intensity of HIF-1α immunolabeling (M) of MGO-treated, uninfected-, or M. tuberculosis-infected BMM determined 24 h after infection. Differences are significant at *, *P* ≤ 0.05 and ***, *P* ≤ 0.001, one-way ANOVA with Welch’s adjustment. (N) The relative HRE activity in RAW macrophages treated or not with MGO was evaluated 24 h after BCG infection. The mean relative luciferase levels ± SEM in triplicate independent cultures are depicted. Differences are significant at **, *P* ≤ 0.01, one-way ANOVA with Welch’s correction.

Methylglyoxal (MGO), a highly reactive α-oxoaldehyde and dicarbonyl formed as a by-product of glycolysis, is increased in the setting of high glucose in DM (35). Whether MGO hampers the HIF-regulated responses of M. tuberculosis- or BCG-infected BMM and the intracellular growth of M. tuberculosis was then analyzed. MGO was toxic for M. tuberculosis-infected or -uninfected BMM at 1,000 μM but not at 750 μM or lower concentrations as determined by LDH release assay or live/dead staining (Fig. S4A and B). We found that incubation of BCG-infected BMM with MGO reduced HIF-regulated *vegfa*, *il1b*, and *inos* transcripts (Fig. 4A to C), as well as the levels of nitrite in culture supernatants (Fig. 4D). Treatment with MGO also reduced inflammatory and metabolic transcripts levels in M. tuberculosis-infected BMM (Fig. 4E to G). Similar to observations in BCG-infected cells, the levels of lactate were increased in M. tuberculosis-infected BMM compared to controls and were decreased in MGO-treated BMM (Fig. 4H). The incubation of BMM with MGO did not modify the uptake of M. tuberculosis (Fig. S4C) but resulted in higher intracellular bacterial levels and frequencies of infected BMM at 5 days after infection (Fig. 4I to K). The bacterial levels of IFN-γ-treated BMM increased when coincubated with MGO albeit displaying lower M. tuberculosis levels than BMM incubated with MGO alone (Fig. 4I to K). The uptake of M. tuberculosis in IFN-γ-treated BMM treated or not with MGO was similar (Fig. S4C).

10.1128/mbio.01086-22.4 FIG S4 (A and B) BMM were incubated with the indicated concentrations of MGO and infected 4 h later with BCG. The mean % lactate dehydrogenase in the culture supernatants ± SEM and the % of live BMM ± SEM determined by flow cytometry after incubation with live/dead staining are depicted. Differences with untreated BMM are significant at *, *P* ≤ 0.05; **, *P* ≤ 0.01; and ***, *P* ≤ 0.001, one-way ANOVA. (C) IFN-γ-treated and control BMM were treated with 400 μM MGO 4 h before infection with M. tuberculosis*-*gfp (MOI 1:1). Representative histogram overlays of M. tuberculosis-gfp determined 4 h after infection are shown. Download FIG S4, EPS file, 0.3 MB. Copyright © 2022 Terán et al. 2022 Terán et al. https://creativecommons.org/licenses/by/4.0/ This content is distributed under the terms of the Creative Commons Attribution 4.0 International license.

Despite the reduced HIF-regulated transcripts and the increased intracellular growth of M. tuberculosis in MGO-treated BMM, MGO treatment of BMM increased the HIF-1α protein levels when measured before and after infection with M. tuberculosis (Fig. 4L and M), suggesting that the reduced levels of HIF-1-regulated transcripts are not due the destabilization of HIF-1α. However, the transcriptional activity of HIF-1, as measured by a luciferase reporter, was reduced by incubation with 400 μM MGO before or after BCG infection of macrophages (Fig. 4N).

### Impaired HIF-1-responses and M. tuberculosis control in high-glucose-treated BMM and in hyperglycemic *Lepr^db/db^* mice *in vivo*.

**FIG 5:**
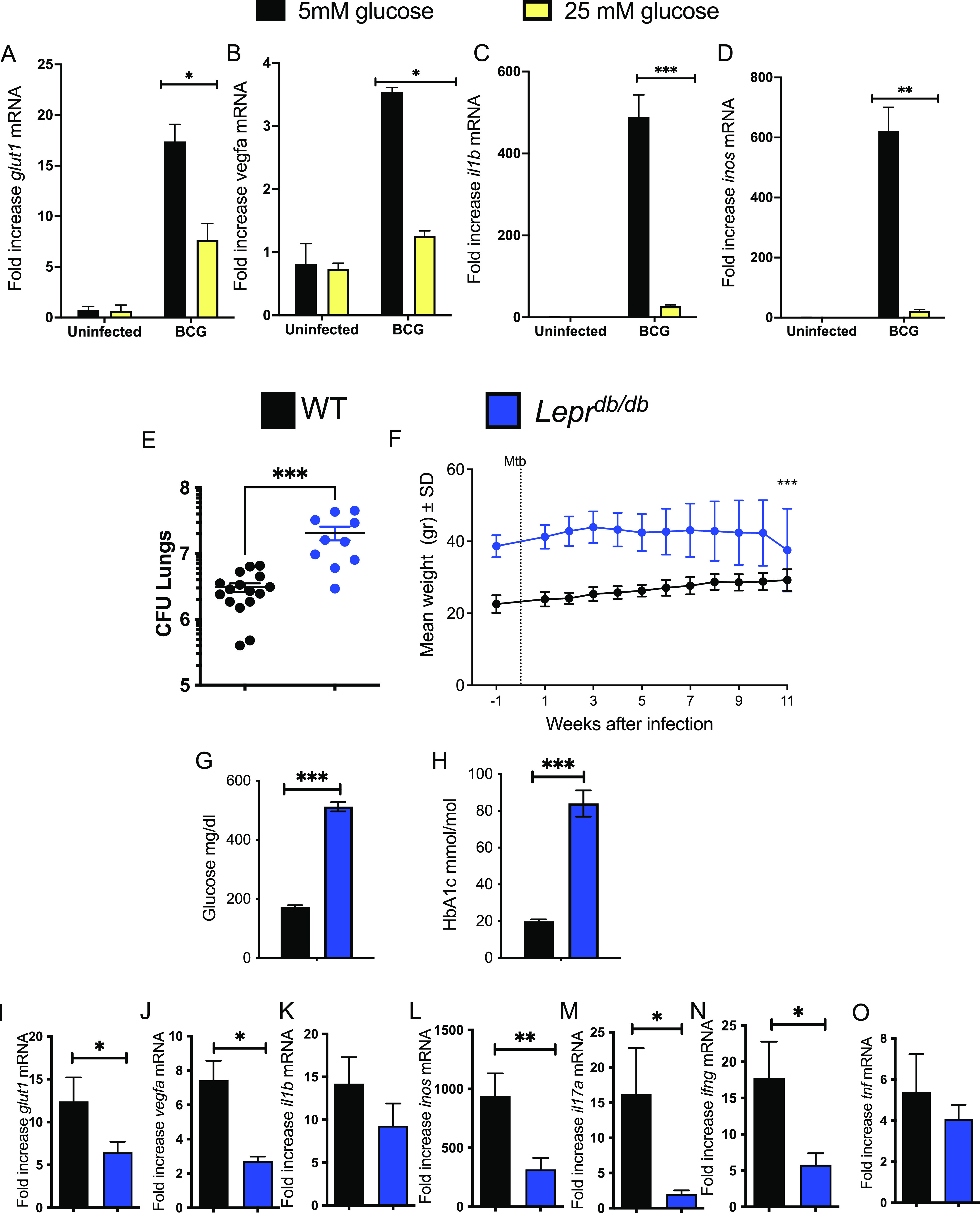
Impaired HIF-1-responses and enhanced susceptibility to M. tuberculosis infection in hyperglycemic *Lepr^db/db^* mice and in high-glucose-level-treated BMM. (A to D) BMM were medium containing 5 mM or 25 mM glucose and infected with BCG 3 days after. Total RNA was extracted 24 h after infection and the levels of *glut1* (A), *vegfa* (B), *il1b* (C), and *inos* (D) and *hrpt* were measured by real-time PCR. The mean fold increase of transcript levels in triplicate cultures ± SEM is depicted. One out of at least 2 independent experiments is depicted. (E) *Lepr^db/db^* and control C57BL/6 mice were sacrificed 11 weeks after aerosol infection with M. tuberculosis, and CFU (CFU) per lung were assessed. The CFU per organ of individual mice and the median per group at the indicated time points after infection are depicted (median CFU/lung *Lepr^db/db^*: 20.7·10^6^; WT: 3.08·10^6^). Differences in CFU are significant (***, *P* < 0.001, Mann-Whitney U test). (F) The mean weight ± SEM of *Lepr^db/db^* and WT mice during infection with M. tuberculosis (*n* = 9 animals per group). Differences between groups were significant at ***, *P* ≤ 0.001, two-way ANOVA test. (G and H) The levels of glucose (G) and HbA1c (H) were measured in the plasma of infected WT and *Lepr^db/db^* mice 11 weeks after infection with M. tuberculosis as detailed in Materials and Methods. Differences between WT and *Lepr^db/db^* groups are significant at ***, *P* < 0.001, unpaired Student's *t* test. (I to O) Total RNA was extracted from the lungs of *Lepr^db/db^* and control C57BL/6 mice 11 weeks after aerosol infection with M. tuberculosis. The levels of *glut1* (I), *vegfa* (J), *il1b* (K), *inos* (L), *il17a* (M), *ifng* (N), and *tnf* (O) mRNA were determined by real-time PCR. The mean fold increase of mRNA level ± SEM in 8 mice per group of 1 of 2 independent experiments is depicted. Differences between groups are significant (*, *P* < 0.05; **, *P* < 0.01; ***, *P* < 0.001, unpaired Student's *t* test with Welch’s correction).

We then studied whether incubation of mycobacteria-infected BMM in a high-glucose concentration altered the accumulation of HIF-1-dependent transcripts. For this purpose, BMM were cultured in either 5 mM or 25 mM glucose for 3 days before infection with BCG. We observed that levels of *glut1*, *vegfa*, *il1b*, and *inos* transcripts were all reduced in mycobacteria-infected BMM when cultured in 25 mM compared to those cultured in 5 mM glucose (Fig. 5A to D).

Leptin receptor-deficient *Lepr^db/db^* mice develop significant obesity, fasting hyperglycemia, and hyperinsulinemia and are a model for type 2 DM (36). *Lepr^db/db^* mice showed enhanced M. tuberculosis loads in lungs when measured 12 weeks postinfection (Fig. 5E). As expected, *Lepr^db/db^* were weightier and showed higher levels of blood glucose and HbA1c than C57BL/6 controls before and after M. tuberculosis infection that were compatible with those that characterize DM (Fig. 5F to H). HbA1c levels were higher in *Lepr^db/db^* mice after infection with M. tuberculosis (Fig. 5H).

Immune and metabolic HIF-1-regulated transcripts were reduced in lungs from M. tuberculosis-infected *Lepr^db/db^* compared to wild-type (WT) mice (Fig. 5I to L). Moreover, *ifng* and *il17a* transcripts were reduced while *tnf* mRNA levels (that are not regulated by HIF-1) were similar in mutant and WT*-*M. tuberculosis-infected mice (Fig. 5M to O).

### DFO reverts the MGO-hampered HIF-1-mediated responses and the enhanced susceptibility to M. tuberculosis infection of BMM.

**FIG 6:**
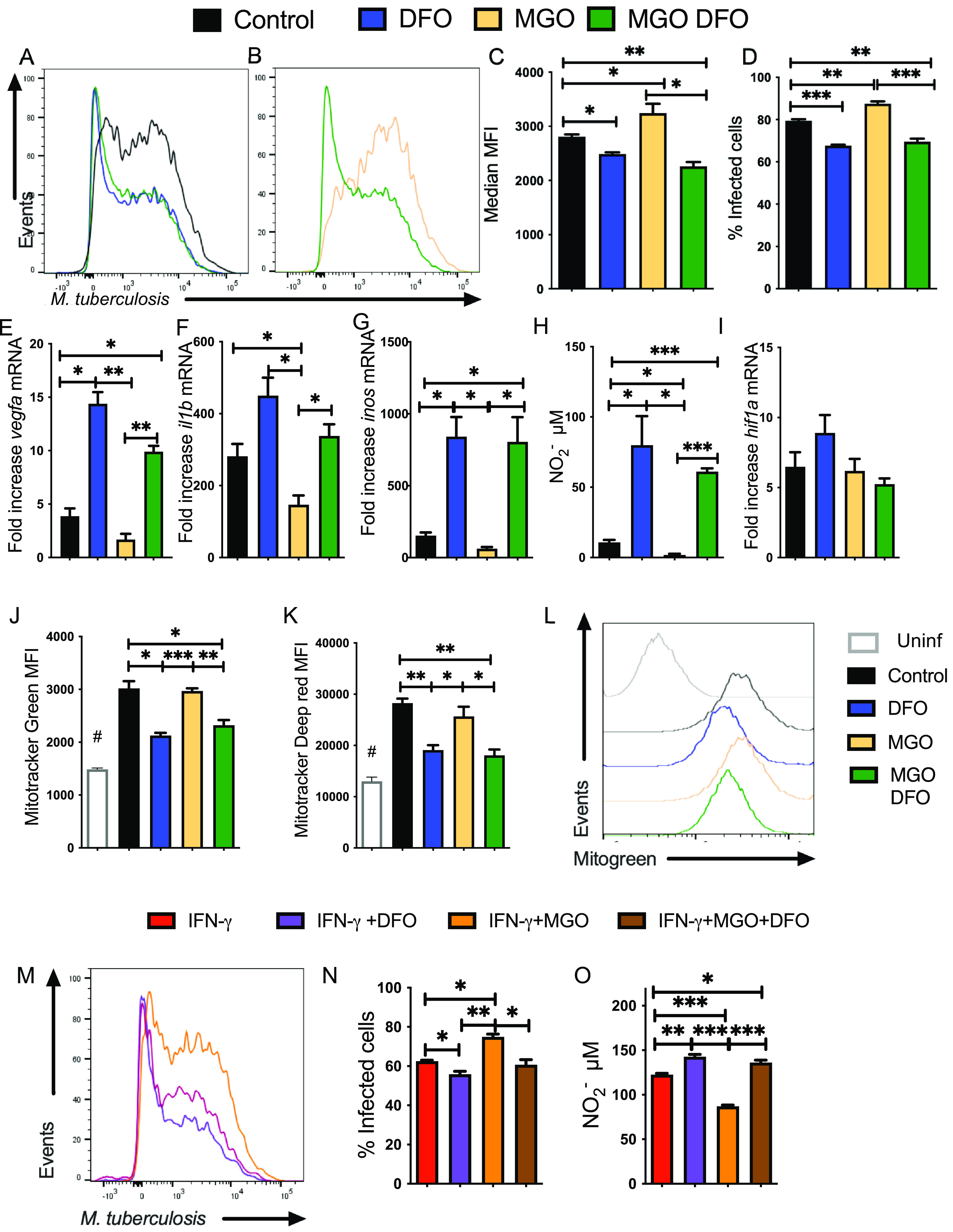
DFO reverts the MGO-hampered HIF-1-mediated responses and the enhanced susceptibility to M. tuberculosis infection of BMM. (A to D) A group of BMM cultures were treated with MGO. Four hours later, MGO-treated and control BMM were infected with M. tuberculosis*-*gfp (MOI 1:1). BMM were either treated with 100 μM DFO or left untreated 4 h after infection. Representative histograms (A and B), the mean M. tuberculosis-gfp MFI (C), and the mean percentage of infected BMM (D) at 5 days after infection are shown. Differences are significant at *, *P* ≤ 0.05; **, *P* ≤ 0.01; and ***, *P* ≤ 0.001, one-way ANOVA test with Welch’s adjustment. (E to G and I) BMM were incubated with MGO and 1 h after being infected with M. tuberculosis. MGO-treated or control BMM were further incubated with DFO 4 h after infection. The mean fold increase of *vegfa* (E), *il1b* (F), *inos* (G), and *hif1a* (I) mRNA was measured in triplicate BMM cultures ± SEM was measured by RT-PCR. Differences are significant at *, *P* ≤ 0.05 and **, *P* ≤ 0.01, one-way ANOVA test with Welch’s correction. (H) The mean concentration of nitrite ± SEM in supernatants of MGO and/or DFO-treated, M. tuberculosis-infected BMM was measured 72 h after culture. Differences are significant at *, *P* ≤ 0.05 and ***, *P* ≤ 0.001, one-way, Welch’s adjusted, ANOVA test. (J to L) BMM were either treated with MGO 4 h before BCG infection and/or with DFO 4 h after infection. A group of BMM was left uninfected. Forty-eight hours after infection, BMM were incubated with Mito tracker Green (I) or Mito tracker Deep Red (J) for 30 min at 37°C. The mean fluorescence intensity of the different groups (*n* = 3 per group) ± SEM and representative histograms of Mito Tracker Green-labeled cells (K) are shown. This experiment was repeated three times. Differences are significant at *, *P* ≤ 0.05, **, *P* ≤ 0.01; and ***, *P* ≤ 0.001, one-way Welch’s adjusted ANOVA test. #, Differences with the uninfected group are statistically significant. (M and N) BMM was treated with IFN-γ 48 h before infection with M. tuberculosis. Further, a group of BMM was treated with MGO 4 h before infection. MGO-treated and control BMM were infected with M. tuberculosis*-*gfp. Four hours after infection, BMM were either treated with 100 μM DFO or left untreated. Representative histograms (M) and the mean percentage of infected BMM ± SEM (N) quantified 5 days after infection are shown. Differences are significant at *, *P* ≤ 0.05 and **, *P* ≤ 0.01, one-way ANOVA test with Welch’s correction. (O) The nitrite concentration in supernatants of IFN-γ-activated BMM treated with MGO and/or DFO treated was measured 72 h after infection with M. tuberculosis. Differences are significant at *, *P* ≤ 0.05; **, *P* ≤ 0.01; and ***, *P* ≤ 0.001, one-way Welch ANOVA test.

We then tested whether DFO was able to revert the MGO-impaired metabolic and inflammatory responses and M. tuberculosis control. The frequency of infected cells as well as the intracellular bacterial loads was reduced by coincubation of DFO in MGO-treated BMM to levels similar to those registered in DFO-treated BMM (Fig. 6A to D). *vegfa*, *il1b*, and *inos* transcripts, as well as nitrite concentration in M. tuberculosis-infected BMM, were reduced when treated with MGO, but the addition of DFO restored these responses to levels similar to those determined in DFO-treated M. tuberculosis-infected BMM (which were, in turn, higher than untreated controls) (Fig. 6E to H). In agreement, *inos* mRNA and nitrite levels in BCG-infected BMM, MGO-treated BMM were restored by incubation with DFO (Fig. S5A and B).

10.1128/mbio.01086-22.5 FIG S5 DFO restores HIF-1-mediated responses in MGO treated BCG-infected BMM. (A) BMM were incubated with MGO and infected with BCG 1 h after treatment. MGO treated or control BMM were further incubated with DFO 4 h after infection. The mean fold increase of *inos* mRNA were measured in triplicate BMM cultures ± SEM was measured by RT-PCR. (B) The concentration of nitrite in the supernatants of MGO- and/or DFO-treated BMM was measured 72 h after infection with BCG. (A and B) Differences are significant at *, *P* ≤ 0.05 and **, *P* ≤ 0.01, one-way Welch’s adjusted ANOVA test. Download FIG S5, EPS file, 0.1 MB. Copyright © 2022 Terán et al. 2022 Terán et al. https://creativecommons.org/licenses/by/4.0/ This content is distributed under the terms of the Creative Commons Attribution 4.0 International license.

As shown above, DFO and MGO controlled the protein levels of HIF-1α and HIF-1 activity in BMM; however, *hif1a* mRNA accumulation in M. tuberculosis-infected BMM treated with either DFO and/or MGO or left untreated was similar (Fig. 6I).

HIF-1 mediates the activation of the glycolytic pathway and decreased flux through the tricarboxylic acid cycle, to decrease mitochondrial reactive oxygen species (ROS) production (37). BMM were loaded with MitoTracker Green or MitoTracker Deep Red probes to detect mitochondrial content and mitochondrial membrane potential, respectively. We found that the mitochondrial content and potential membrane were increased after mycobacterial infection of BMM. The levels of Mitotracker Green and Deep red were reduced in mycobacteria-infected BMM treated with DFO as well as those treated with MGO and DFO, in comparison to untreated, infected controls or to infected BMM incubated with MGO (Fig. 6J to L).

The incubation with DFO also reduced the percentage of M. tuberculosis-infected BMM that were stimulated with IFN-γ and treated with MGO. The percentage of IFN-γ activated, DFO- and MGO-treated-infected BMM was similar to that of IFN-γ-stimulated (DFO- and MGO-untreated) BMM (Fig. 6M and N).

IFN-γ stimulated M. tuberculosis*-*infected BMM showed a higher level of nitrite in the supernatant compared to nonstimulated-infected BMM (Fig. 6O). The addition of DFO further increased nitrite levels whereas incubation with MGO diminished nitrite concentration by IFN-γ-activated-infected BMM. The nitrite levels in IFN-γ-activated M. tuberculosis-infected BMM treated with MGO and DFO were similar to those of IFN-γ-stimulated, -untreated BMM (Fig. 6O).

### DFO reverts the high-glucose-impaired HIF-1-mediated responses and M. tuberculosis control by BMM.

**FIG 7:**
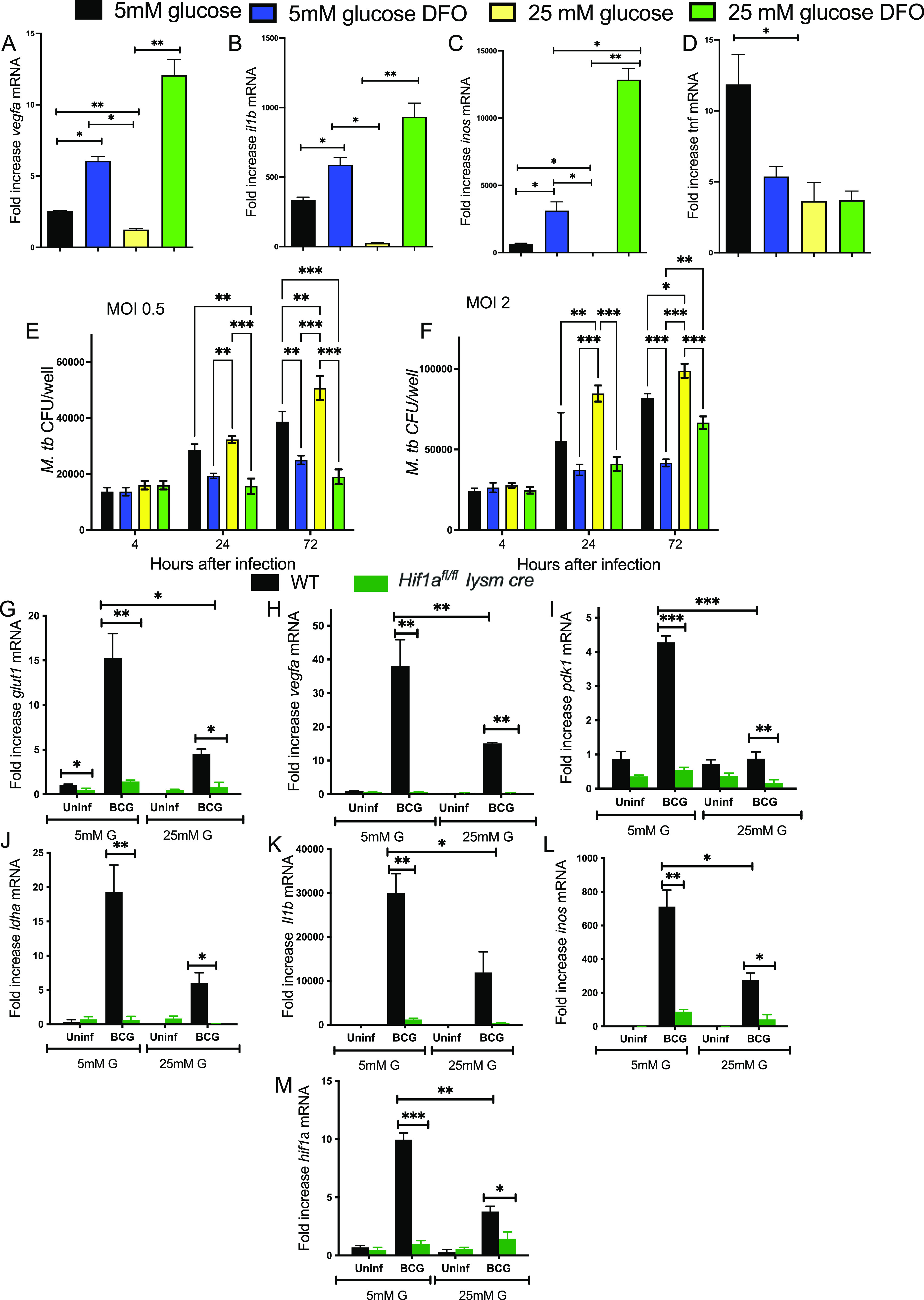
DFO reverts the high-glucose concentration-hampered HIF-1-mediated responses and the enhanced susceptibility to M. tuberculosis infection of BMM. (A to D) BMM were cultured in medium containing 5 mM or 25 mM glucose and infected with BCG 3 days after. DFO was added 4 h after infection in indicated groups. Total RNA was extracted 24 h after infection and the levels of *vegfa* (A), *il1b* (B), *inos* (C), and *tnf* (D) mRNA in triplicate independent cultures were measured by real-time PCR. The mean fold increase of transcript levels ± SEM is depicted. Differences between groups are significant at *, *P* ≤ 0.05; **, *P* ≤ 0.01; and ***, *P* ≤ 0.001, one-way ANOVA test with Welch’s correction. (E and F) BMM were incubated with 5 mM or 25 mM glucose 3 days before infection with M. tuberculosis at MOI 0.5 (E) or 2:1 (F). BMM were treated with 100 μM DFO 4 h after the infection. The CFU in cell lysates at the indicated time points after infection was determined. The mean CFU ± SEM in triplicate independent cultures per condition is shown. Differences between groups are significant at **, *P* ≤ 0.05 and **, *P* ≤ 0.01, one-way ANOVA test with Welch’s correction. (G to M) *Hif1a^fl/fL^ lysm cre* and WT BMM were infected with BCG at an MOI 5:1. Total RNA was extracted 24 h after infection and the levels of *glut1* (G), *vegfa* (H), *pdk1* (I), *ldha* (J), *il1b* (K), *inos* (L), and *hif1a* (M) mRNA in triplicate independent cultures were in either 5 mM or 25 mM glucose measured by real-time PCR. The mean fold increase of transcript levels in triplicate cultures ± SEM is depicted. Differences are significant at *, *P* ≤ 0.05; **, *P* ≤ 0.01; and ***, *P* ≤ 0.001, one-way ANOVA test with Welch’s correction.

Next, the effect of DFO on BCG-infected BMM incubated at normal or high-glucose levels was evaluated. We found that the reduced *vegfa*, *il1b*, and *inos* mRNA levels in infected BMM cultured in high-glucose concentrations were restored by coincubation with DFO (Fig. 7A to C), while levels of *tnf* were not altered by the treatments (Fig. 7D).

Whether high-glucose concentrations could alter the control of M. tuberculosis growth in BMM was then tested. BMM were infected with M. tuberculosis at an MOI 0.5 and MOI 2, and DFO was added 4 h after. BMM cultured in normal or high-glucose levels showed similar uptake of M. tuberculosis at 4 h after infection (Fig. 7E and F). The BMM cultured in 25 mM glucose showed higher titers of M. tuberculosis than BMM cultured in 5 mM glucose. DFO treatment reduced M. tuberculosis bacterial numbers at 72 h after infection compared to those in nontreated BMM. Coincubation with DFO also reduced M. tuberculosis titers in 25 mM glucose-treated BMM (Fig. 7E and F).

To confirm that HIF-1 is a main transcriptional controller of inflammatory and metabolic genes during mycobacterial infection, the responses of *Hif1a*-deficient (*Hif1a^fl/fL^ lysm cre*) and control BMM to infection were studied. *Vegfa*, *glut-1*, *pdk1*, and *ldha* mRNA were reduced in BCG-infected and noninfected *Hif1a^fl/fL^ lysm cre* BMM compared to controls (Fig. 7G to J). Moreover, *il1b* and *inos* mRNA levels were also diminished in *hif1a*-deficient BMM (Fig. 7K and L). The levels of metabolic and immune transcripts in mycobacteria-infected *Hif1a^fl/fL^ lysm cre* BMM incubated in high and normal glucose conditions were similar (Fig. 7G to L). As a control, *Hif1a* mRNA levels were reduced after infection of *Hif1a^fl/fL^ lysm cre* BMM (Fig. 7M). Altogether, this suggests that loss of HIF-1 abrogates the metabolic and immune responses to infection with mycobacteria.

## DISCUSSION

**FIG 8:**
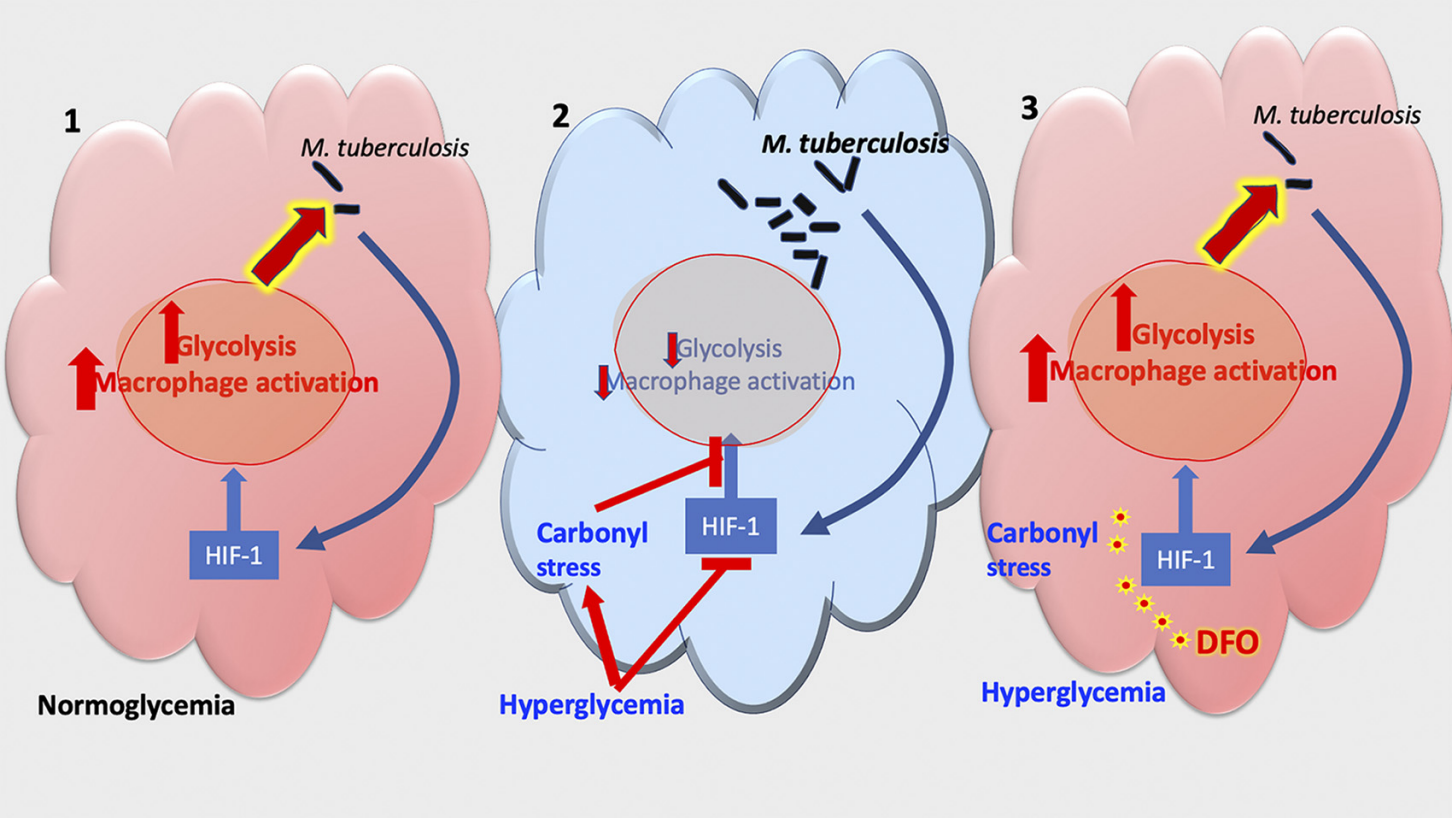
Summary. (1) Infection with M. tuberculosis induces the expression of HIF-1-regulated genes after in BMM. These genes mediate the metabolic switch and macrophage activation required to control the intracellular bacterial infection. (2) During DM, high levels of glucose and reactive carbonyl compounds hamper the expression of HIF-1-regulated genes and in turn impair the control of intracellular M. tuberculosis in BMM. (3) Treatment with the hypoxia mimic deferoxamine (DFO) increased immunometabolic responses in infected BMMs and in the lungs of M. tuberculosis-infected mice. DFO treatment reduced the M. tuberculosis titers in BMM treated or not with IFN-γ. Treatment with DFO restored HIF-1-regulated responses to infection and improved M. tuberculosis control in MGO and high-glucose-treated BMM.

Here, we showed that chemical stabilization of HIF-1 by DFO increased the metabolic and immune activation and improved bacterial control during M. tuberculosis infection *in vitro* and *in vivo*. High-glucose concentration and MGO treatment reduced HIF-1-dependent immunometabolic responses and diminished the intracellular M. tuberculosis control in BMM. Moreover, the stabilization or increased transcriptional activation of HIF-1 by DFO restored both BMM activation and the restriction of intracellular bacterial growth in BMM treated with MGO or incubated in high-glucose conditions (Fig. 8).

Specifically, we showed that BMM infected with M. tuberculosis or BCG and lungs from M. tuberculosis-infected mice had increased levels of HIF-1α and HIF-1-regulated immune and metabolic transcripts as well as NO release. HIF-1 expression in immune cells can be triggered by hypoxia but also by inflammation and stimulation by infectious microorganisms in an oxygen-independent manner (38). Bacteria-induced expression of HIF-1α has been shown in macrophages cultured under normoxic conditions in the presence of different pathogens or innate immune receptor ligands (39, 40), as also shown here for virulent M. tuberculosis or the attenuated BCG mycobacterial strains.

The incubation with DFO further increased the HIF-1 levels in BMMs, the glycolytic metabolism, as shown by increased levels of lactate and transcripts for key glycolytic enzyme transcripts, as well as reduced mitochondrial functions. Our data confirm previous results using Seahorse technology, showing that DFO enhances glycolytic metabolism in M. tuberculosis-stimulated human macrophages during mitochondrial distress (41). DFO has also been shown to improve the efficiency of antibiotic treatment of BCG-infected macrophages (42). While DFO may display a direct antimycobacterial effect (43), bacterial toxicity was not observed at the DFO concentrations we used. DFO also supported the augmented expression of HIF-regulated inflammatory genes in M. tuberculosis*-*infected BMM. Administration of DFO to uninfected or M. tuberculosis-infected mice increased their HIF-1-regulated responses and resulted in a reduction of the levels of M. tuberculosis in lungs. The reduction of bacterial load in DFO-treated animals was moderate, supporting the possibility of testing other HIF stabilizers as adjunctive TB treatment or improving DFO delivery. Distinct responses to HIF stabilization in different cell populations or the relatively short half-life of DFO *in vivo* (44) could also explain the moderate protection against M. tuberculosis after DFO administration *in vivo*. Instead, BMM incubated with DFO showed an important reduction of intracellular M. tuberculosis levels. DFO further reduced bacterial load in IFN-γ-activated BMM, in agreement with an increased NO production. DFO has been indicated to impair HIF-1α hydroxylation by inhibiting the activity of iron-dependent prolyl hydroxylases (45–47). In addition to HIF-1α stability, O_2_ and iron levels also regulate the transactivation activity of HIF-1α, which is mediated by factor inhibiting HIF-1 (FIH-1), which hydroxylates an asparagine residue of HIF-1α and inhibits HIF-1α activity by limiting recruitment of the coactivator CBP/p300 (48). DFO has been shown to be an effective inhibitor of FIH, promoting full transcriptional activity of HIF-1 (49). Despite the important association with HIF-1 as a target mechanism for DFO, whether other intracellular mechanisms regulated by iron chelation could account for the improved mycobacterial control by DFO cannot be ruled out by our study. For example, the intracellular availability of iron is crucial for bacterial growth and iron regulation via ferropontin and hepcidin might also affect infection control by DFO (50).

Destabilization or functional repression of HIF-1 is the event that transduces hyperglycemia into the loss of the cellular response to hypoxia in DM (30, 48, 51).

MGO, a ubiquitous product of cellular metabolism, is the most significant and highly reactive glycating agent *in vivo* and is considered one of the critical factors in the pathogenesis of diabetic complications (5). The primary source for MGO synthesis in eukaryotic cells is triose phosphates from the glycolytic bypass. As triose phosphates are intermediate products of glycolysis, blood and intracellular glucose levels are determinants of MGO levels. Most of the effects of carbonyl compounds are executed by AGEs, which bind to and alter the tertiary structure and function of proteins. Several studies have reported the positive association between plasma carbonyl compounds and AGEs levels in DM patients (52). While MGO has been associated with oxidative and inflammatory responses through the formation of AGEs (52), here we showed that MGO decreases the expression of HIF-1-regulated immunometabolic molecules and impairs the control of M. tuberculosis during infection of IFN-γ activated and control BMM. Of interest, MGO did not decrease the levels of HIF-1α in BMM before or after infection with M. tuberculosis but might have inhibited the expression of HIF-1α-regulated genes by a ROS-mediated modification of p300 (31, 48, 53, 54). In line with this, HIF-1 activity, not stability, has been shown to be impaired in a high-glucose environment due to decreased association of HIF-1α and its coactivator p300 (30, 55). In agreement, BMM cultured in high-glucose conditions also showed diminished HIF-1-regulated genes and infected BMM cultured in high-glucose levels showed higher intracellular M. tuberculosis levels than BMM cultured in normoglycemic conditions.

The leptin receptor-deficient *Lepr*^db/db^ diabetic mice showed increased susceptibility to M. tuberculosis infection, confirming previous observations using *Lepr*^db/db^ as well as in leptin-deficient *Lep^ob/ob^* mice (56, 57). *Lepr*^db/db^ mice showed reduced levels of expression of HIF-1-controlled inflammatory and metabolic genes during infection with M. tuberculosis, which associates with the impaired responses of M. tuberculosis*-*infected BMM treated with MGO and high-glucose levels *in vitro*. Direct leptin sensing in immune cells has been shown to be dispensable for immune control of M. tuberculosis *in vivo* (56).

Promoting HIF-1 function with DFO was able to revert the inhibition of immunometabolic responses and the defective intracellular bacterial control by both high-glucose and MGO treatment in control and IFN-γ-activated macrophages. In line with this, maintaining HIF-1 signaling has been shown to prevent DM complications in patients as well as in experimental models (32, 51, 58–61). The deletion of *hif1a* gene in BMM decreased metabolic and inflammatory transcripts as well as metabolites, confirming a nonredundant role of HIF-1 in the activation of these genes that cannot be compensated by HIF-2.

Diabetes increases the risk of several microbial infections, including those by viruses such as Sars-CoV-2, influenza, hepatitis B, and hepatitis C and bacteria such as Streptococcus pneumoniae, Helicobacter pylori, Staphylococcus aureus, *Haemophylus influenza*, and Escherichia coli among others that affect the respiratory tract and the urinary tract or cause soft tissue infections (such as foot infections) (62). The inhibition of HIF-responses in DM may also regulate the risk of acquiring or developing an increased severity of these infections.

Altogether, our results suggest repressed HIF-1-mediated responses in DM as a mechanism underlying the increased risk of developing TB in DM patients. We also show that promoting HIF-1 function with DFO was able to revert the inhibition of immunometabolic responses and the defective intracellular bacterial control driven by both high-glucose and MGO treatment in control and IFN-γ activated macrophages, constituting a rationale for the design of adjunctive TB therapy in TB/DM comorbidity.

## MATERIALS AND METHODS

### Ethics statement.

The animals were housed and handled at Astrid Fagreus Laboratory, Karolinska Institutet, Stockholm, according to directives and guidelines of the Swedish Board of Agriculture, the Swedish Animal Protection Agency, and the Karolinska Institute (djurskyddslagen 1988:534; djurskyddsförordningen 1988:539; and djurskyddsmyndigheten DFS 2004:4). The study was performed under approval of the Stockholm North Ethical Committee on Animal Experiments permits no. N128/16. Animals were housed under specific pathogen-free conditions.

### Mice.

Six-week-old BKS(D)-*Lepr*db/JOrlRj (*Lepr^db/db^*) obese leptin receptor-deficient mice and C57BL/6 mice were purchased from Janvier (Le Genest St. Isle, France).

All animals used in this study were males between 8 to 20 weeks of age. The animals were housed in groups (three or five) and given free access to food and water.

The mice were under weekly follow-up for body weight and blood glucose. All procedures were performed in a biosafety level III animal facility.

Macrophages from *Hif1a^fl/fL^ Lysm cre* mice were used for *in vitro* experiments. *Hif1a^fl/fL^* mice with a loxP-targeted deletion of the *hif1a* and the *vhl* gene (19, 63) were crossed with *Lysm cre* transgenic animals in which cre expression is driven by the lysozyme M promoter (64), allowing the deletion of the transcription factor in the myeloid lineage.

### Regents.

Deferoxamine (DFO), 2-deoxyglucose (2-DG), and d-glucose (Sigma-Aldrich, St. Louis, MO) were used at indicated concentrations for *in vitro* and *in vivo* experiments. DFO was diluted in PBS and freshly prepared before inoculation. Mice in the DFO group were treated i.p. with a 400-mg/kg dose every other day starting 1 day before infection and during 3 months of M. tuberculosis infection until 1 day before take down. Similar DFO treatment of mice schemes was previously reported to lack toxicity (65).

### Infection of mice with M. tuberculosis.

Mice were infected with 200 CFU M. tuberculosis Harlingen strain using a nose-only aerosol exposure unit (In-tox Products, NM, USA). The dose indicates the bacteria recovered in lungs 24 h after infection. A 15-ml suspension of 1 × 10^6^ M. tuberculosis per ml was loaded into a nebulizer, and animals inhaled the bacteria aerosol for 20 min. Mice were sacrificed at indicated time points after infection and bacteria from organ lysates was plated and quantified on Middlebrook 7H11 agar containing 10% enrichment of oleic acid, albumin, dextrose, catalase, 5 μg amphotericin B per ml, and 8 μg/ml polymyxin B grown for 3 weeks at 37°C.

### Measurement of blood glucose, HbA1c, triglycerides, and cholesterol.

A quantitative determination of cholesterol, triglycerides, and glucose levels was determined in 10 μl tail vein blood using the Multicare-in device (Balerna, Switzerland). Blood glucose was measured every week, while triglycerides and cholesterol were measured every 2 weeks until mice take down.

Glycated hemoglobin (HbAc1) was measured using the DCA Vantage Analyzer (Siemens) device in 1 μl tail vein blood. HbA1c measurements were done before and after infection with M. tuberculosis.

### Generation of mouse bone marrow-derived macrophages.

Bone marrow cells were flushed from tibia and femurs with PBS, filtered through a 70-μm cell strainer, resuspended in DMEM supplemented with 10% FCS and 30% L929 cell-conditioned medium (as a source of macrophage-colony-stimulating factor), and incubated for 6 days at 37°C, 5% CO_2_. Bone marrow-derived macrophage (BMM) cultures were then washed with PBS and detached with 1% trypsin, and 5·10^5^ cells were seeded to each well at (using 24-well plates). BMM were further incubated for 24 h at 37°C before infections or treatment with diverse compounds. Confirming previous data (66), BMM were F4/80^+^, CD11b^+^.

### Infection of BMM with mycobacteria.

M. tuberculosis Harlingen or H37Rv carrying the green fluorescent protein (GFP)-encoding pFPV2 plasmid were grown in Middlebrook 7H9 (Difco, Detroit, MI) supplemented with albumin, dextrose, and catalase and quantified by densitometry. BMM were infected with sonicated bacteria at a multiplicity of infection (MOI) of 0.5 and 2. After 4 h, cells were washed twice with PBS to remove extracellular bacteria and further incubated for 1 to 5 days. At these time points, BMM were lysed with 0.1% Triton X, and the suspensions were plated on Difco Middlebrook 7H11 agar enriched with oleic acid-albumin-dextrose catalase supplement (BD) to quantify M. tuberculosis CFU.

Alternatively, BMM were infected as described above at different MOI (0.3, 1, or 3) with M. tuberculosis-GFP (67). The infected BMM were detached using trypsin-EDTA at different times after infection. Cell suspensions were incubated with live/dead stain (LIVE/DEAD Fixable Yellow Dead Cell Stain, Invitrogen), washed with FACS buffer (PBS containing 0.5% FCS and 0.5 mM EDTA), and fixed with 4% formaldehyde (Sigma-Aldrich) at room temperature (RT) for 10 min. Data were acquired on a LSRII flow cytometer and analyzed with FlowJo software (Tree star Inc., Ashland, OR). Cells in the mononuclear gate were ≥95% live, and ≥95% of live cells were CD11b^+^, F4/80^+^ (see the gating strategy in Fig. S6).

10.1128/mbio.01086-22.6 FIG S6 Gating strategy used for quantification of intracellular M. tuberculosis-gfp. Gating strategy for flow cytometry experiments assessing single cycle infectivity. FSC-A versus SSC-A was used to gate nucleated cells; FSC-A and FSC-H were used to gate single cells and exclude doublets and live cells were selected by a live/dead staining and most of cells expressed CD11b and F4/80. The M. tuberculosis GFP^+^ population was plotted versus FSC-A. The dot plots show M. tuberculosis-gfp expression in BMM 4 h after infection. Download FIG S6, JPG file, 0.2 MB. Copyright © 2022 Terán et al. 2022 Terán et al. https://creativecommons.org/licenses/by/4.0/ This content is distributed under the terms of the Creative Commons Attribution 4.0 International license.

### Immunofluorescent labeling of HIF-1α.

HIF-1α was labeled in 4% PFA-fixed M. tuberculosis-infected or uninfected BMM treated or not with DFO or MGO. Cells were stained with anti-HIF-1α (1:100; clone HIF-1α67; BD) antibody in 1:1 ratio of 1× PBS with 0.1% Tween 20 (PBS-T) and 5% BSA and 5% donkey serum in PBS. Cells were washed with 1× PBS-T and further incubated with secondary Alexa Fluor 488-conjugated goat anti-mouse IgG antibody (Invitrogen) 1:100 for 1 h at room temperature, followed by staining with DAPI for 15 min and washed and mounted in Fluoromount G (Southern Biotech). Slides were analyzed with a Leica epi-fluorescence microscope using 40× objective. Fluorescence intensity was quantified in three to five random fields of view per coverslip with the Cell Profiler software pipeline. Briefly, DAPI channel was segmented using the “IdentifyPrimaryObjects” function to detect nuclei, and Rhodamine Red intensity was then measured.

### Mitochondrial content and potential.

M. tuberculosis-infected BMM were detached and stained with 150 nM MitoTracker Green fluorescent dye or with MitoTracker Deep Red (Thermo Fisher Scientific) for 30 min 37C. The far red-fluorescent MitoTracker Deep Red was used to measure the mitochondrial membrane potential while Mitotracker green labeling was used as an estimation of the mitochondrial mass. Fluorescence-labeled mitochondria in BMM were fixed with subjected after fixation to FACS analysis.

### l-lactate assay.

Lactate accumulation in BMM supernatants was measured using the l-lactate assay kit (Cayman Chemicals) as described in the manufacturer’s protocol. In the assay, lactate dehydrogenase catalyzes the oxidation of lactate to pyruvate, along with the concomitant reduction of NAD^+^ to NADH. NADH reacts with the fluorescent substrate to yield a highly fluorescent product. The fluorescent product was analyzed after a 15-min RT incubation with BMM supernatants with an excitation wavelength of 540 nm.

### Nitrite.

To analyze the concentration of the stable oxidation products of nitric oxide (NO) in the BMM supernatants, the total concentration of nitrite and nitrite was calculated by performing the Griess reaction as previously described (68). Briefly, 100 μl of 1% (wt/vol) sulfanilamide in 5% phosphoric acid followed by 100 μl 0.1% (wt/vol) *N*-(1-naphtyl) ethylenediamine HCl was added to 50 μl of samples. After incubation for 10 min at RT, the absorbance was read at 540 nm.

### Real-time PCR.

Total RNA was extracted from lung samples or culture cells using TRIzol (Sigma-Aldrich) and cDNA was obtained by reverse transcription. Transcripts were quantified by real-time PCR as previously described (69). The relative number of transcripts was calculated using the 2^−(ΔΔ^*^CT^*^)^ method. These values were then used to calculate the relative expression of mRNA in the different conditions (infection and/or treatment) used in tissues and cells. Transcripts were quantified using *hprt* as a control housekeeping gene to calculate the Δ*CT* values for individual samples.

The primer sequences for sense (S) and antisense (AS) were as follows: *hprt* (S) 5′-CCCAGCGTCGTGATTAGC-3′ and *hprt* (AS) 5′-GGAATAAACACTTTTTCCAAATCC-3′; *inos* (S) 5′-CAGCTGGGCTGTACAAACCTT-3′ and *inos* (AS) 5′-CATTGGAAGTGAAGCGTTTCG-3′; *il1b* (S) 5′-TGGTGTGTGACGTTCCCATT-3′ and *il1b* (AS) 5′-CAGCACGAGGCTTTTTTGTTG-3′; *tnf* (S) 5′-GGCTGCCCCGACTACGT-3′ and *tnf* (AS) 5′-GACTTTCTCCTGGTATGAGATAGCAAA-3′; *ifng* (S) 5′-GCT TTG CAG CTC TTC CTC AT-3′ and *ifng* (AS) 5′-CAC ATC TAT GCC ACT TGA GTT AAA ATA GT-3′; *glut1* (S) 5′-AAGTCCAGGAGGATATTCAG-3′ and *glut1* (AS) 5′-CTACAGTGTGGAGATAGGAG-3′; *vegfa* (S) 5′-TAGAGTACATACTTCAAGCCG-3′ and *vegfa* (AS) 5′-TCTTTCTTTGGTCTGCATTC-3′; *hif1a* (S) 5′-CGATGACACAGAAACTGAAG-3′ and *hif1a* (AS) 5′-GAAGGTAAAGGAGACATTGC-3′; *pdk1* (S) 5′-GAAGCAGTTCCTGGACTTCG -3′ and *pdk1* (AS) 5′-CCAATTTGCACCAGCTGTA -3′; and *ldha* (S) 5′-TGGCAGACTTGGCTGACAG-3′ and *ldha* (AS) 5′-ACCTTCACAACATCCGAGATTC-3′.

### HRE-driven luciferase promoter assay.

HIF-1 activity was determined by an HRE-driven luciferase reporter assay as described previously (58). Briefly RAW macrophages were transiently transfected with HRE-luciferase reporter gene plasmid (pT81/HRE-luc) using Lipofectamine (Thermo Fisher Scientific) according to the manufacturer's instructions. Renilla luciferase vector, which provides constitutive expression, was cotransfected with HRE-luciferase plasmid and used as an internal control. Transfected cells were infected or not with BCG (24 h after transfection) and cultured in media containing either DFO or MGO for 24 h. The macrophages were harvested, and luciferase activity was measured using the Dual Luciferase Assay System (Promega) on the GloMax Luminometer (Promega) according to the manufacturer’s instructions. HRE-driven firefly luciferase activity was normalized to Renilla luciferase activity and expressed as relative luciferase activity.

### Statistical analysis.

All *in vitro* assays were performed at least in biological triplicates and were independently twice or more. Differences in bacterial counts in the lungs of infected mice were calculated using the nonparametric Mann-Whitney’s U test. Differences in cytokine transcripts, lactate, nitrites, and CFU were measured by unpaired Student's *t* test considering unequal variances (Welch’s test) and by one-way ANOVA using Welch’s adjustment when comparing three or more groups. In this case, a two-way ANOVA was used when more than two parameters were analyzed (i.e., time after infection and treatments). Multiple comparisons were corrected by the false discovery rate method. The statistical tests were performed using the Prism software (GraphPad, La Jolla, CA).
