# Supplementary figures and images for: High Glucose and Carbonyl Stress Impair HIF-1-Regulated Responses and the Control of Mycobacterium tuberculosis in Macrophages
Source: mBio. 2022 Sep 19;13(5):e01086-22. doi: 10.1128/mbio.01086-22 (PMC9600926; doi:10.1128/mbio.01086-22)

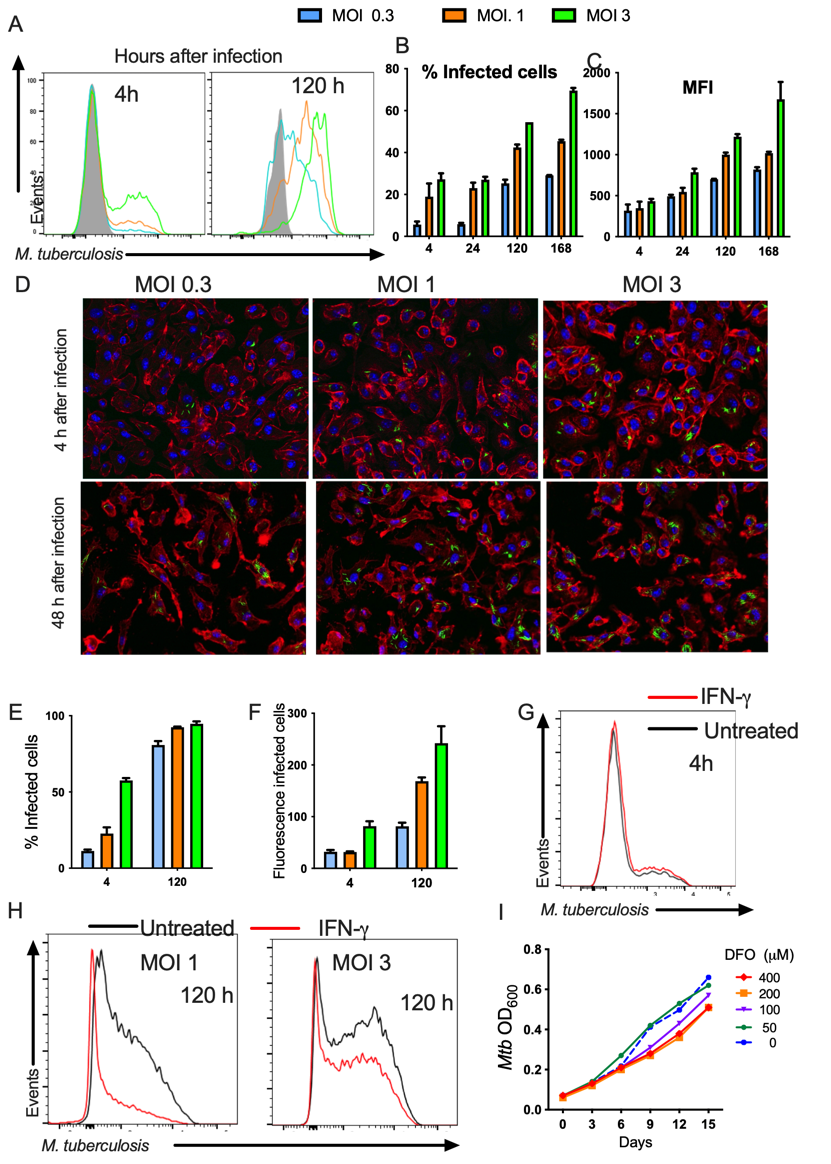

Supplement: FIG S3 [file mbio.01086-22-s0003.tif]

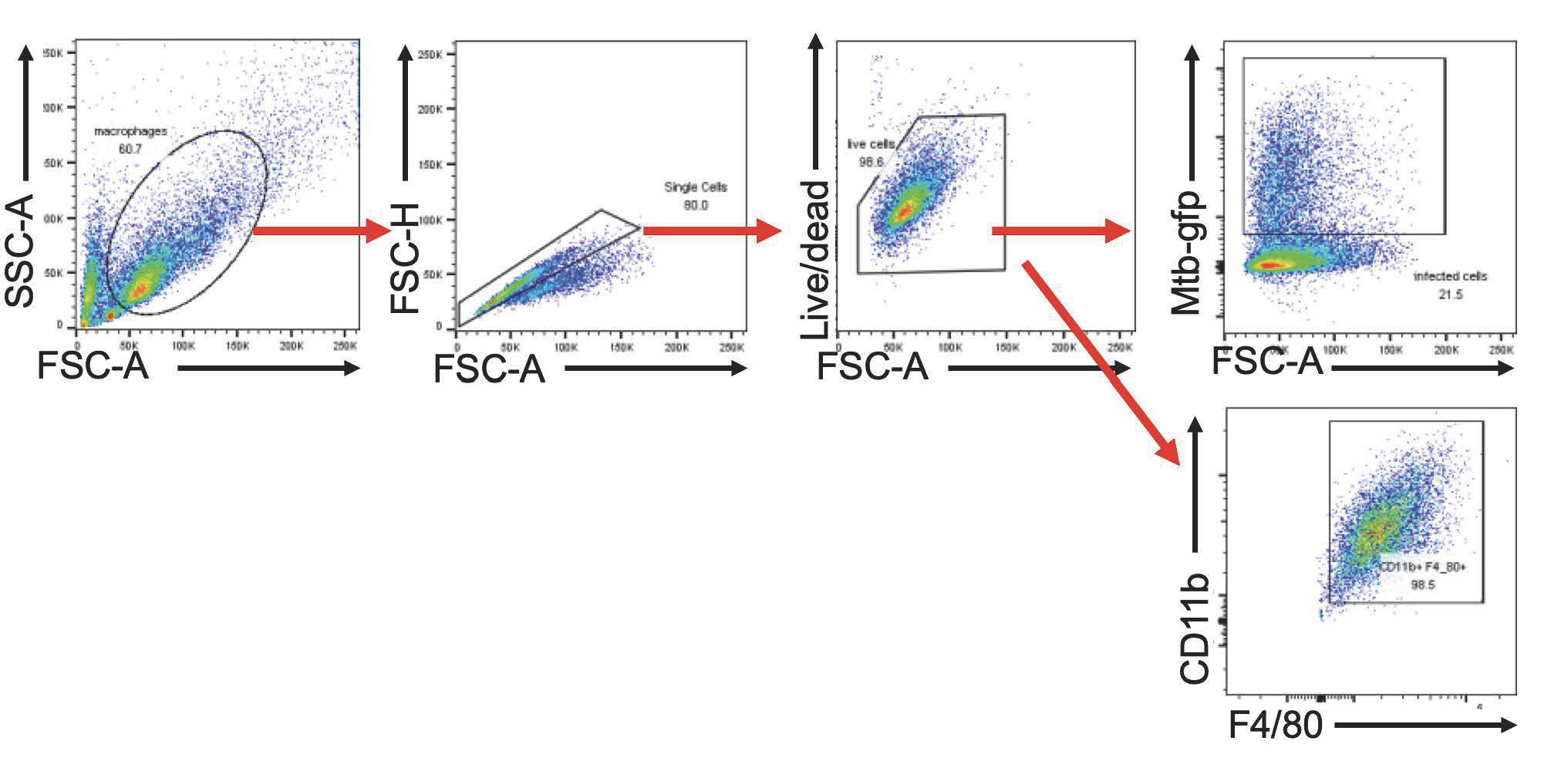

Supplement: FIG S6 [file mbio.01086-22-s0006.jpg]
